# 8β-Acet­oxy-14α-benzo­yloxy-*N*-methyl-13β,15α-dihy­droxy-1α,6α,16β-trimeth­oxy-4β-(meth­oxy­meth­yl)aconitane: hypaconitine isolated from ‘fuzi’

**DOI:** 10.1107/S1600536810039887

**Published:** 2010-10-09

**Authors:** Ling-Li Zheng, Yan Li, Tie-Ying Zi, Ming-Yong Yuan

**Affiliations:** a The First Affiliated Hospital, Chengdu Medical College, Xindu 610500, People’s Republic of China

## Abstract

The title compound, C_33_H_45_NO_10_, has an aconitine carbon skeleton with four six-membered rings and two five-membered rings. The five-membered rings adopt envelope configurations and the six-membered N-containing heterocyclic ring displays a chair conformation. Two intra­molecular O—H⋯O hydrogen bonds occur.

## Related literature

The title compound is an aconitine-type C_19_-diterpenoid alkaloid, which is isolated from the roots of *Aconitum carmichaeli* Debx., known as fuzi. For reviews of diterpenoid alkaloids, see Wang *et al.* (2009 ▶, 2010 ▶); Wang & Chen (2010 ▶). For the chemical structure of the title compound established from NMR and MS data, see: Pelletier *et al.* (1984 ▶). For the crystal structures of related C_19_-diterpenoid alkaloids, see: Gao *et al.* (2010 ▶); Tashkhodjaev & Sultankhodjaev (2009 ▶); He *et al.* (2008 ▶). For the absolute configuration of aconitine-type diterpenoid alkaloids, see: Pelletier & Djarmati (1976 ▶); Tsuda & Marion (1963 ▶); Zhapova *et al.* (1986 ▶).
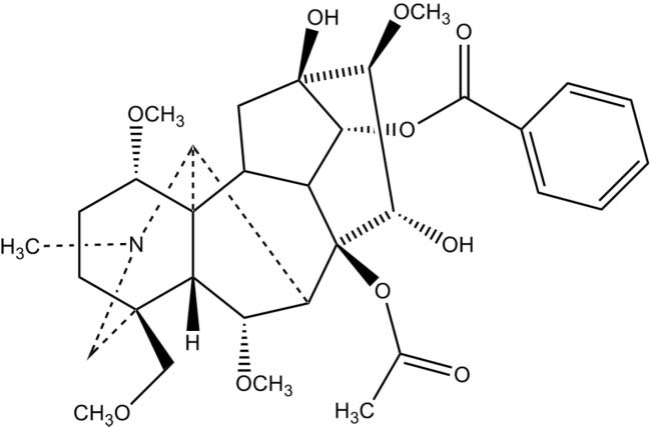


## Experimental

### 

#### Crystal data

C_33_H_45_NO_10_
*M* *_r_* = 615.70
Orthorhombic, 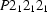

*a* = 12.457 (2) Å
*b* = 15.689 (3) Å
*c* = 15.771 (3) Å
*V* = 3082.3 (10) Å^3^
*Z* = 4
Mo *K*α radiation
μ = 0.10 mm^−1^
*T* = 113 K
0.32 × 0.29 × 0.21 mm

#### Data collection

Rigaku Saturn CCD area-detector diffractometer
Absorption correction: multi-scan (*CrystalClear*; Rigaku/MSC, 2005 ▶) *T*_min_ = 0.969, *T*_max_ = 0.980
28410 measured reflections
4094 independent reflections
3805 reflections with *I* > 2σ(*I*)
*R*_int_ = 0.040

#### Refinement

*R*[*F* ^2^ > 2σ(*F* ^2^)] = 0.035
*wR*(*F* ^2^) = 0.083
*S* = 1.02
4094 reflections
406 parameters
3 restraints
H-atom parameters constrained
Δρ_max_ = 0.25 e Å^−3^
Δρ_min_ = −0.21 e Å^−3^

### 

Data collection: *CrystalClear* (Rigaku/MSC, 2005 ▶); cell refinement: *CrystalClear*; data reduction: *CrystalClear*; program(s) used to solve structure: *SHELXS97* (Sheldrick, 2008 ▶); program(s) used to refine structure: *SHELXL97* (Sheldrick, 2008 ▶); molecular graphics: *ORTEP03* (Farrugia, 1997 ▶); software used to prepare material for publication: *SHELXL97*.

## Supplementary Material

Crystal structure: contains datablocks I, global. DOI: 10.1107/S1600536810039887/ng5034sup1.cif

Structure factors: contains datablocks I. DOI: 10.1107/S1600536810039887/ng5034Isup2.hkl

Additional supplementary materials:  crystallographic information; 3D view; checkCIF report

## Figures and Tables

**Table 1:** Hydrogen-bond geometry (Å, °)

*D*—H⋯*A*	*D*—H	H⋯*A*	*D*⋯*A*	*D*—H⋯*A*
O8—H8⋯O5	0.84	2.11	2.7887 (19)	137
O10—H10*O*⋯O9	0.84	2.05	2.5620 (19)	118

**Fig. 1.**
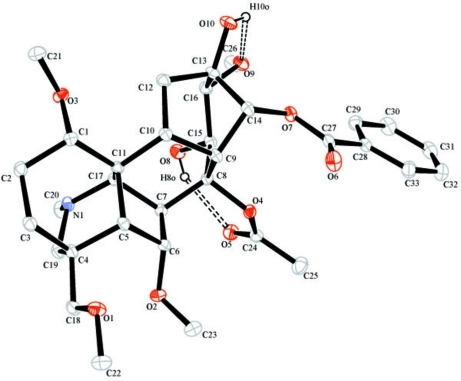
Perspective view of the title compound.

**Fig. 2.**
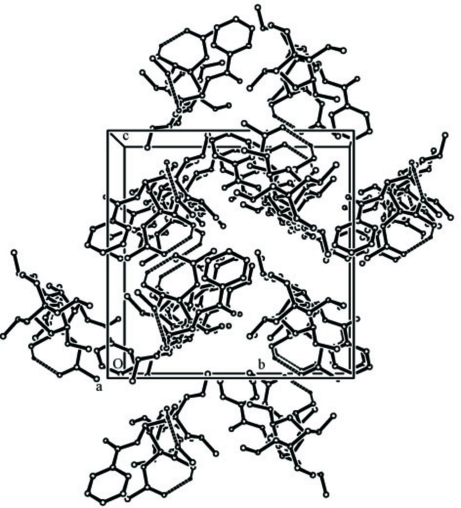
View of the packing of the title compoun

## supplementary crystallographic information

### Comment

As a famous Chinese traditional herbal, the plant *Aconitum carmichaeli* Debx. known as fuzi, has been therapeutically used to the treatment of rheumatic pain, rheumatoid arthritis and some other inflammations. The title compound 8β-acetoxy- 14α-benzoyloxy-*N*-methyl-13β,15α-dihydroxy-1α,6α,16β-trimethoxy-4β-(methoxymethylene)aconitane, hypaconitine, has been isolated previously from A. carmichaeli Debx. (Pelletier *et al.* 1984), and its structure was established from the NMR and MS data. However, the crystal structure of hypaconitine has not been reported. In view of this, the crystal structure determination of the title compound was carried out and the results are presented here.

The molecular structure of the title compound is shown in Fig. 1. Six-membered ring A (C1/C2/C3/C4/C5/C11) and B (C7/C8/C9/C10/C11/C17) adopt chair conformations; six-membered heterocyclic ring E (C4/C5/C11/C17/N1/C19) adopts the same chair conformation; the five-membered rings C (C9/C10/C12/C13/C14) and F (C5/C6/C7/C17/C11) display an envelope conformation. While the six-membered ring D (C8/C9/C14/C13/C16/C15) adopt boat conformations. The crystal structure contains intermolecular O—H···O hydrogen bond between the hydroxy group and carbonyl O atom. The absolute configuration of the title compound can not be confirmed by the MoKa diffraction data. But it could be determined throngh the comparion of the similar natural products for their unique and same configuration (He *et al.*, 2008).

### Experimental

The title compound was isolated from the roots of Aconitum carmichaeli Debx. according to the literature procedure of Gao *et al.* (2010) and crystals of X-ray quality were grown from acetone at room temperature by slow evaporation.

### Refinement

Hydroxy H atoms were located in a difference Fourier map and refined as riding in their as-found relative positions with *U*_iso_(H) = 1.5*U*_eq_(O). Other H atoms were located geometrically with C—H = 0.93–0.98 Å, and refined using a riding model with *U*_iso_(H) = 1.5*U*_eq_(C) for methyl and 1.2*U*_eq_(C) for the others. The absolute configuration has not been determined from the X-ray analysis, owing to the absence of strong anomalous scattering, and Friedel's pairs were merged. Bond distance restraints for three bonds were applied. The absolute configuration was assigned on the basis of the related literature (Pelletier & Djarmati, 1976; Tsuda & Marion, 1963; Zhapova *et al.*, 1986).

### Crystal data

**Table:**

C_33_H_45_NO_10_	*F*(000) = 1320
*M**_r_* = 615.70	*D*_x_ = 1.327 Mg m^−^^3^
Orthorhombic, *P*2_1_2_1_2_1_	Mo *K*α radiation, λ = 0.71073 Å
Hall symbol: P 2ac 2ab	Cell parameters from 11109 reflections
*a* = 12.457 (2) Å	θ = 1.3–27.9°
*b* = 15.689 (3) Å	µ = 0.10 mm^−^^1^
*c* = 15.771 (3) Å	*T* = 113 K
*V* = 3082.3 (10) Å^3^	Block, colourless
*Z* = 4	0.32 × 0.29 × 0.21 mm

### Data collection

**Table:**

Rigaku Saturn CCD area-detector diffractometer	4094 independent reflections
Radiation source: rotating anode	3805 reflections with *I* > 2σ(*I*)
multiplayer	*R*_int_ = 0.040
Detector resolution: 14.63 pixels mm^-1^	θ_max_ = 27.9°, θ_min_ = 1.8°
ω and φ scans	*h* = −16→12
Absorption correction: multi-scan (*CrystalClear*; Rigaku/MSC, 2005)	*k* = −20→20
*T*_min_ = 0.969, *T*_max_ = 0.980	*l* = −20→20
28410 measured reflections	

### Refinement

**Table:**

Refinement on *F*^2^	Primary atom site location: structure-invariant direct methods
Least-squares matrix: full	Secondary atom site location: difference Fourier map
*R*[*F*^2^ > 2σ(*F*^2^)] = 0.035	Hydrogen site location: inferred from neighbouring sites
*wR*(*F*^2^) = 0.083	H-atom parameters constrained
*S* = 1.02	*w* = 1/[σ^2^(*F*_o_^2^) + (0.0542*P*)^2^] where *P* = (*F*_o_^2^ + 2*F*_c_^2^)/3
4094 reflections	(Δ/σ)_max_ < 0.001
406 parameters	Δρ_max_ = 0.25 e Å^−^^3^
3 restraints	Δρ_min_ = −0.21 e Å^−^^3^

### Special details

**Table:**

Geometry. All e.s.d.'s (except the e.s.d. in the dihedral angle between two l.s. planes) are estimated using the full covariance matrix. The cell e.s.d.'s are taken into account individually in the estimation of e.s.d.'s in distances, angles and torsion angles; correlations between e.s.d.'s in cell parameters are only used when they are defined by crystal symmetry. An approximate (isotropic) treatment of cell e.s.d.'s is used for estimating e.s.d.'s involving l.s. planes.
Refinement. Refinement of *F*^2^ against ALL reflections. The weighted *R*-factor *wR* and goodness of fit *S* are based on *F*^2^, conventional *R*-factors *R* are based on *F*, with *F* set to zero for negative *F*^2^. The threshold expression of *F*^2^ > σ(*F*^2^) is used only for calculating *R*-factors(gt) *etc*. and is not relevant to the choice of reflections for refinement. *R*-factors based on *F*^2^ are statistically about twice as large as those based on *F*, and *R*- factors based on ALL data will be even larger.

### Fractional atomic coordinates and isotropic or equivalent isotropic displacement parameters (Å^2^)

**Table:**

	*x*	*y*	*z*	*U*_iso_*/*U*_eq_	Occ. (<1)
O1	1.23858 (11)	0.89466 (8)	0.31289 (9)	0.0302 (3)	
O2	1.07598 (11)	0.84457 (8)	0.13939 (9)	0.0302 (3)	
O3	0.91073 (10)	0.62972 (8)	0.42341 (8)	0.0256 (3)	
O4	0.79646 (10)	0.87205 (8)	0.12467 (8)	0.0224 (3)	
O5	0.80028 (12)	0.78668 (9)	0.00869 (8)	0.0314 (3)	
O6	0.66939 (11)	1.02025 (8)	0.23958 (11)	0.0381 (4)	
O7	0.60560 (10)	0.88599 (8)	0.23532 (8)	0.0245 (3)	
O8	0.74141 (11)	0.66278 (8)	0.12664 (9)	0.0278 (3)	
H8	0.7649	0.6749	0.0782	0.042*	
O9	0.52663 (10)	0.71641 (9)	0.22035 (8)	0.0286 (3)	
O10	0.55459 (10)	0.76257 (9)	0.37461 (9)	0.0302 (3)	
H10O	0.5011	0.7562	0.3428	0.045*	
N1	1.03362 (12)	0.64061 (9)	0.23693 (10)	0.0228 (3)	
C1	0.98310 (14)	0.69978 (11)	0.41053 (11)	0.0216 (4)	
H1	0.9735	0.7401	0.4591	0.026*	
C2	1.09733 (14)	0.66559 (12)	0.41484 (12)	0.0252 (4)	
H2A	1.1154	0.6519	0.4744	0.030*	
H2B	1.1023	0.6124	0.3814	0.030*	
C3	1.17712 (15)	0.72968 (12)	0.38090 (12)	0.0254 (4)	
H3A	1.2493	0.7033	0.3798	0.030*	
H3B	1.1800	0.7792	0.4198	0.030*	
C4	1.14877 (14)	0.76100 (12)	0.29144 (11)	0.0226 (4)	
C5	1.04062 (14)	0.80980 (11)	0.29511 (11)	0.0213 (4)	
H5	1.0466	0.8612	0.3323	0.026*	
C6	0.99822 (14)	0.83478 (12)	0.20478 (11)	0.0222 (4)	
H6	0.9574	0.8894	0.2099	0.027*	
C7	0.91787 (14)	0.76308 (11)	0.18088 (11)	0.0208 (4)	
H7	0.9326	0.7409	0.1226	0.025*	
C8	0.80427 (15)	0.80051 (11)	0.18664 (11)	0.0206 (4)	
C9	0.79423 (14)	0.84829 (11)	0.27182 (11)	0.0210 (4)	
H9	0.8295	0.9054	0.2679	0.025*	
C10	0.84333 (14)	0.79591 (11)	0.34650 (11)	0.0217 (4)	
H10	0.8581	0.8372	0.3934	0.026*	
C11	0.95087 (14)	0.74792 (11)	0.32833 (11)	0.0197 (4)	
C12	0.74725 (14)	0.73924 (12)	0.37588 (12)	0.0258 (4)	
H12A	0.7630	0.6783	0.3653	0.031*	
H12B	0.7339	0.7471	0.4373	0.031*	
C13	0.64957 (14)	0.76738 (12)	0.32456 (11)	0.0238 (4)	
C14	0.67831 (14)	0.85795 (12)	0.30017 (11)	0.0231 (4)	
H14	0.6738	0.8963	0.3507	0.028*	
C15	0.70879 (14)	0.73879 (11)	0.16864 (11)	0.0224 (4)	
H15	0.6605	0.7694	0.1282	0.027*	
C16	0.63726 (15)	0.71180 (12)	0.24422 (12)	0.0240 (4)	
H16	0.6546	0.6514	0.2593	0.029*	
C17	0.93745 (14)	0.69281 (11)	0.24888 (11)	0.0209 (4)	
H17	0.8726	0.6556	0.2544	0.025*	
C18	1.24163 (15)	0.81852 (12)	0.26341 (13)	0.0267 (4)	
H18A	1.3109	0.7889	0.2719	0.032*	
H18B	1.2344	0.8325	0.2025	0.032*	
C19	1.13661 (15)	0.68636 (12)	0.22852 (12)	0.0254 (4)	
H19A	1.1429	0.7087	0.1700	0.031*	
H19B	1.1961	0.6456	0.2376	0.031*	
C20	1.02178 (17)	0.57792 (12)	0.16930 (13)	0.0306 (4)	
H20A	0.9539	0.5472	0.1765	0.046*	
H20B	1.0816	0.5374	0.1716	0.046*	
H20C	1.0219	0.6070	0.1143	0.046*	
C21	0.89680 (16)	0.60786 (13)	0.50979 (12)	0.0308 (4)	
H21A	0.9656	0.5893	0.5336	0.046*	
H21B	0.8444	0.5615	0.5144	0.046*	
H21C	0.8707	0.6576	0.5412	0.046*	
C22	1.32008 (17)	0.95307 (13)	0.29058 (14)	0.0355 (5)	
H22A	1.3905	0.9271	0.3007	0.053*	
H22B	1.3130	1.0047	0.3251	0.053*	
H22C	1.3133	0.9679	0.2305	0.053*	
C23	1.0854 (9)	0.9162 (7)	0.0954 (9)	0.058 (3)*	0.333 (16)
H23A	1.0189	0.9267	0.0639	0.087*	0.333 (16)
H23B	1.1454	0.9109	0.0555	0.087*	0.333 (16)
H23C	1.0990	0.9637	0.1342	0.087*	0.333 (16)
C23'	1.0983 (3)	0.9342 (2)	0.1242 (3)	0.0289 (12)*	0.667 (16)
H23D	1.1498	0.9396	0.0775	0.043*	0.667 (16)
H23E	1.1286	0.9598	0.1756	0.043*	0.667 (16)
H23F	1.0315	0.9636	0.1092	0.043*	0.667 (16)
C24	0.79618 (15)	0.85677 (13)	0.04047 (12)	0.0267 (4)	
C25	0.78886 (19)	0.93861 (14)	−0.00785 (14)	0.0385 (5)	
H25A	0.7831	0.9263	−0.0686	0.058*	
H25B	0.8534	0.9728	0.0026	0.058*	
H25C	0.7254	0.9704	0.0107	0.058*	
C26	0.49317 (17)	0.65471 (14)	0.15989 (14)	0.0386 (5)	
H26A	0.5280	0.6662	0.1054	0.058*	
H26B	0.4151	0.6577	0.1530	0.058*	
H26C	0.5134	0.5977	0.1796	0.058*	
C27	0.61137 (15)	0.96722 (11)	0.20869 (13)	0.0255 (4)	
C28	0.53567 (14)	0.98128 (12)	0.13698 (12)	0.0254 (4)	
C29	0.47300 (16)	0.91484 (13)	0.10516 (12)	0.0292 (4)	
H29	0.4788	0.8593	0.1287	0.035*	
C30	0.40216 (18)	0.93033 (14)	0.03905 (14)	0.0370 (5)	
H30	0.3594	0.8853	0.0173	0.044*	
C31	0.39364 (18)	1.01139 (15)	0.00471 (13)	0.0383 (5)	
H31	0.3456	1.0216	−0.0409	0.046*	
C32	0.45470 (18)	1.07705 (15)	0.03665 (15)	0.0415 (5)	
H32	0.4483	1.1327	0.0134	0.050*	
C33	0.52578 (16)	1.06205 (13)	0.10295 (15)	0.0353 (5)	
H33	0.5677	1.1075	0.1249	0.042*	

### Atomic displacement parameters (Å^2^)

**Table:**

	*U*^11^	*U*^22^	*U*^33^	*U*^12^	*U*^13^	*U*^23^
O1	0.0263 (7)	0.0297 (7)	0.0346 (8)	−0.0064 (6)	0.0061 (6)	−0.0036 (6)
O2	0.0296 (7)	0.0332 (7)	0.0277 (7)	−0.0010 (6)	0.0076 (6)	0.0077 (6)
O3	0.0288 (7)	0.0261 (6)	0.0218 (6)	−0.0049 (5)	−0.0031 (5)	0.0017 (5)
O4	0.0232 (6)	0.0241 (6)	0.0198 (6)	0.0034 (5)	−0.0013 (5)	0.0009 (5)
O5	0.0372 (8)	0.0343 (8)	0.0226 (7)	0.0021 (6)	0.0010 (6)	−0.0028 (6)
O6	0.0319 (8)	0.0268 (7)	0.0556 (10)	−0.0007 (6)	−0.0138 (7)	−0.0073 (7)
O7	0.0217 (6)	0.0249 (6)	0.0269 (7)	0.0033 (5)	−0.0036 (5)	−0.0022 (5)
O8	0.0304 (7)	0.0277 (6)	0.0254 (7)	0.0018 (6)	0.0020 (6)	−0.0085 (6)
O9	0.0212 (6)	0.0337 (7)	0.0311 (7)	−0.0025 (6)	−0.0012 (5)	−0.0059 (6)
O10	0.0196 (6)	0.0444 (8)	0.0267 (7)	0.0003 (6)	0.0049 (5)	−0.0018 (6)
N1	0.0244 (8)	0.0211 (7)	0.0228 (7)	0.0049 (6)	−0.0007 (6)	−0.0037 (6)
C1	0.0235 (9)	0.0219 (9)	0.0192 (8)	−0.0019 (7)	−0.0013 (7)	−0.0019 (7)
C2	0.0254 (9)	0.0281 (9)	0.0221 (9)	0.0027 (8)	−0.0041 (7)	0.0021 (8)
C3	0.0208 (9)	0.0307 (10)	0.0248 (9)	0.0026 (8)	−0.0024 (7)	−0.0013 (8)
C4	0.0200 (8)	0.0257 (9)	0.0222 (9)	0.0028 (7)	−0.0009 (7)	−0.0005 (8)
C5	0.0194 (8)	0.0232 (9)	0.0213 (8)	0.0017 (7)	−0.0001 (7)	−0.0026 (7)
C6	0.0200 (8)	0.0254 (9)	0.0212 (8)	0.0023 (7)	0.0014 (7)	0.0015 (7)
C7	0.0229 (9)	0.0215 (8)	0.0181 (8)	0.0025 (7)	0.0005 (7)	−0.0007 (7)
C8	0.0229 (9)	0.0224 (9)	0.0165 (8)	0.0018 (7)	−0.0002 (7)	0.0013 (7)
C9	0.0183 (8)	0.0231 (9)	0.0216 (8)	0.0018 (7)	−0.0012 (7)	−0.0037 (7)
C10	0.0217 (9)	0.0257 (9)	0.0176 (8)	0.0014 (7)	−0.0006 (7)	−0.0024 (7)
C11	0.0191 (8)	0.0218 (9)	0.0183 (8)	0.0015 (7)	0.0008 (6)	−0.0014 (7)
C12	0.0236 (9)	0.0337 (10)	0.0200 (9)	0.0020 (8)	0.0027 (7)	0.0001 (8)
C13	0.0201 (9)	0.0297 (9)	0.0217 (8)	0.0019 (8)	0.0042 (7)	−0.0035 (8)
C14	0.0200 (8)	0.0291 (9)	0.0201 (8)	0.0033 (7)	−0.0013 (7)	−0.0048 (8)
C15	0.0229 (9)	0.0231 (9)	0.0213 (8)	0.0026 (7)	0.0001 (7)	−0.0035 (7)
C16	0.0222 (9)	0.0261 (9)	0.0238 (9)	0.0014 (7)	0.0000 (7)	0.0000 (8)
C17	0.0207 (9)	0.0224 (9)	0.0195 (8)	0.0014 (7)	−0.0003 (7)	−0.0008 (7)
C18	0.0196 (9)	0.0321 (10)	0.0286 (10)	0.0033 (8)	0.0010 (7)	0.0005 (8)
C19	0.0225 (9)	0.0285 (10)	0.0254 (9)	0.0068 (7)	0.0019 (7)	−0.0025 (8)
C20	0.0350 (11)	0.0281 (10)	0.0288 (10)	0.0074 (9)	−0.0028 (8)	−0.0078 (8)
C21	0.0326 (10)	0.0352 (11)	0.0248 (10)	−0.0043 (9)	0.0039 (8)	0.0032 (8)
C22	0.0319 (11)	0.0380 (11)	0.0365 (11)	−0.0081 (9)	0.0043 (9)	0.0028 (9)
C24	0.0231 (9)	0.0355 (11)	0.0214 (9)	0.0013 (8)	−0.0002 (7)	0.0009 (8)
C25	0.0496 (13)	0.0383 (12)	0.0275 (11)	0.0059 (10)	0.0004 (10)	0.0052 (9)
C26	0.0293 (11)	0.0447 (13)	0.0417 (12)	−0.0089 (10)	−0.0035 (9)	−0.0124 (10)
C27	0.0208 (9)	0.0228 (9)	0.0330 (10)	0.0038 (8)	0.0018 (8)	−0.0047 (8)
C28	0.0206 (9)	0.0280 (10)	0.0275 (9)	0.0045 (7)	0.0021 (7)	−0.0023 (8)
C29	0.0308 (10)	0.0298 (10)	0.0269 (10)	0.0006 (9)	0.0001 (8)	−0.0024 (8)
C30	0.0366 (12)	0.0436 (12)	0.0307 (11)	0.0028 (10)	−0.0063 (9)	−0.0100 (10)
C31	0.0372 (12)	0.0546 (14)	0.0232 (10)	0.0161 (11)	−0.0019 (9)	−0.0016 (10)
C32	0.0402 (13)	0.0408 (13)	0.0436 (13)	0.0111 (11)	0.0004 (10)	0.0114 (11)
C33	0.0302 (11)	0.0290 (10)	0.0467 (13)	0.0015 (9)	−0.0034 (9)	0.0036 (9)

### Geometric parameters (Å, °)

**Table:**

O1—C22	1.412 (2)	C11—C17	1.531 (2)
O1—C18	1.427 (2)	C12—C13	1.527 (3)
O2—C23	1.326 (9)	C12—H12A	0.9900
O2—C6	1.423 (2)	C12—H12B	0.9900
O2—C23'	1.453 (4)	C13—C14	1.515 (3)
O3—C21	1.416 (2)	C13—C16	1.546 (3)
O3—C1	1.436 (2)	C14—H14	1.0000
O4—C24	1.349 (2)	C15—C16	1.547 (2)
O4—C8	1.491 (2)	C15—H15	1.0000
O5—C24	1.210 (2)	C16—H16	1.0000
O6—C27	1.205 (2)	C17—H17	1.0000
O7—C27	1.344 (2)	C18—H18A	0.9900
O7—C14	1.435 (2)	C18—H18B	0.9900
O8—C15	1.423 (2)	C19—H19A	0.9900
O8—H8	0.8400	C19—H19B	0.9900
O9—C26	1.421 (2)	C20—H20A	0.9800
O9—C16	1.430 (2)	C20—H20B	0.9800
O10—C13	1.424 (2)	C20—H20C	0.9800
O10—H10O	0.8400	C21—H21A	0.9800
N1—C20	1.458 (2)	C21—H21B	0.9800
N1—C17	1.463 (2)	C21—H21C	0.9800
N1—C19	1.476 (2)	C22—H22A	0.9800
C1—C2	1.522 (2)	C22—H22B	0.9800
C1—C11	1.553 (2)	C22—H22C	0.9800
C1—H1	1.0000	C23—H23A	0.9800
C2—C3	1.512 (3)	C23—H23B	0.9800
C2—H2A	0.9900	C23—H23C	0.9800
C2—H2B	0.9900	C23'—H23D	0.9800
C3—C4	1.535 (2)	C23'—H23E	0.9800
C3—H3A	0.9900	C23'—H23F	0.9800
C3—H3B	0.9900	C24—C25	1.496 (3)
C4—C18	1.532 (3)	C25—H25A	0.9800
C4—C19	1.542 (2)	C25—H25B	0.9800
C4—C5	1.551 (2)	C25—H25C	0.9800
C5—C6	1.569 (2)	C26—H26A	0.9800
C5—C11	1.571 (2)	C26—H26B	0.9800
C5—H5	1.0000	C26—H26C	0.9800
C6—C7	1.552 (2)	C27—C28	1.489 (3)
C6—H6	1.0000	C28—C33	1.382 (3)
C7—C8	1.535 (2)	C28—C29	1.396 (3)
C7—C17	1.557 (2)	C29—C30	1.387 (3)
C7—H7	1.0000	C29—H29	0.9500
C8—C9	1.543 (2)	C30—C31	1.386 (3)
C8—C15	1.560 (2)	C30—H30	0.9500
C9—C14	1.519 (2)	C31—C32	1.376 (3)
C9—C10	1.561 (2)	C31—H31	0.9500
C9—H9	1.0000	C32—C33	1.390 (3)
C10—C12	1.561 (3)	C32—H32	0.9500
C10—C11	1.563 (2)	C33—H33	0.9500
C10—H10	1.0000		
			
C22—O1—C18	112.81 (15)	C13—C14—H14	110.4
C23—O2—C6	122.1 (5)	C9—C14—H14	110.4
C23—O2—C23'	22.6 (6)	O8—C15—C16	107.08 (14)
C6—O2—C23'	110.7 (2)	O8—C15—C8	112.77 (14)
C21—O3—C1	113.49 (14)	C16—C15—C8	117.95 (14)
C24—O4—C8	120.76 (14)	O8—C15—H15	106.1
C27—O7—C14	118.66 (15)	C16—C15—H15	106.1
C15—O8—H8	109.5	C8—C15—H15	106.1
C26—O9—C16	115.13 (15)	O9—C16—C13	106.42 (14)
C13—O10—H10O	109.5	O9—C16—C15	109.77 (15)
C20—N1—C17	112.88 (14)	C13—C16—C15	114.84 (14)
C20—N1—C19	110.50 (15)	O9—C16—H16	108.5
C17—N1—C19	116.81 (13)	C13—C16—H16	108.5
O3—C1—C2	108.10 (14)	C15—C16—H16	108.5
O3—C1—C11	109.14 (13)	N1—C17—C11	109.39 (14)
C2—C1—C11	116.76 (15)	N1—C17—C7	115.83 (14)
O3—C1—H1	107.5	C11—C17—C7	100.41 (13)
C2—C1—H1	107.5	N1—C17—H17	110.3
C11—C1—H1	107.5	C11—C17—H17	110.3
C3—C2—C1	111.37 (15)	C7—C17—H17	110.3
C3—C2—H2A	109.4	O1—C18—C4	108.36 (15)
C1—C2—H2A	109.4	O1—C18—H18A	110.0
C3—C2—H2B	109.4	C4—C18—H18A	110.0
C1—C2—H2B	109.4	O1—C18—H18B	110.0
H2A—C2—H2B	108.0	C4—C18—H18B	110.0
C2—C3—C4	112.77 (15)	H18A—C18—H18B	108.4
C2—C3—H3A	109.0	N1—C19—C4	113.38 (14)
C4—C3—H3A	109.0	N1—C19—H19A	108.9
C2—C3—H3B	109.0	C4—C19—H19A	108.9
C4—C3—H3B	109.0	N1—C19—H19B	108.9
H3A—C3—H3B	107.8	C4—C19—H19B	108.9
C18—C4—C3	106.26 (14)	H19A—C19—H19B	107.7
C18—C4—C19	109.61 (15)	N1—C20—H20A	109.5
C3—C4—C19	111.77 (15)	N1—C20—H20B	109.5
C18—C4—C5	112.08 (14)	H20A—C20—H20B	109.5
C3—C4—C5	108.89 (14)	N1—C20—H20C	109.5
C19—C4—C5	108.27 (14)	H20A—C20—H20C	109.5
C4—C5—C6	112.45 (14)	H20B—C20—H20C	109.5
C4—C5—C11	109.01 (14)	O3—C21—H21A	109.5
C6—C5—C11	102.57 (13)	O3—C21—H21B	109.5
C4—C5—H5	110.8	H21A—C21—H21B	109.5
C6—C5—H5	110.8	O3—C21—H21C	109.5
C11—C5—H5	110.8	H21A—C21—H21C	109.5
O2—C6—C7	109.96 (14)	H21B—C21—H21C	109.5
O2—C6—C5	117.11 (14)	O1—C22—H22A	109.5
C7—C6—C5	104.86 (14)	O1—C22—H22B	109.5
O2—C6—H6	108.2	H22A—C22—H22B	109.5
C7—C6—H6	108.2	O1—C22—H22C	109.5
C5—C6—H6	108.2	H22A—C22—H22C	109.5
C8—C7—C6	107.64 (14)	H22B—C22—H22C	109.5
C8—C7—C17	112.00 (14)	O2—C23—H23A	109.5
C6—C7—C17	104.17 (14)	O2—C23—H23B	109.5
C8—C7—H7	110.9	O2—C23—H23C	109.5
C6—C7—H7	110.9	O2—C23'—H23D	109.5
C17—C7—H7	110.9	O2—C23'—H23E	109.5
O4—C8—C7	108.02 (13)	H23D—C23'—H23E	109.5
O4—C8—C9	101.52 (13)	O2—C23'—H23F	109.5
C7—C8—C9	108.18 (14)	H23D—C23'—H23F	109.5
O4—C8—C15	107.34 (13)	H23E—C23'—H23F	109.5
C7—C8—C15	117.05 (14)	O5—C24—O4	124.69 (18)
C9—C8—C15	113.46 (14)	O5—C24—C25	124.88 (18)
C14—C9—C8	112.43 (14)	O4—C24—C25	110.43 (17)
C14—C9—C10	101.71 (14)	C24—C25—H25A	109.5
C8—C9—C10	111.67 (14)	C24—C25—H25B	109.5
C14—C9—H9	110.3	H25A—C25—H25B	109.5
C8—C9—H9	110.3	C24—C25—H25C	109.5
C10—C9—H9	110.3	H25A—C25—H25C	109.5
C9—C10—C12	102.90 (14)	H25B—C25—H25C	109.5
C9—C10—C11	116.81 (14)	O9—C26—H26A	109.5
C12—C10—C11	115.92 (14)	O9—C26—H26B	109.5
C9—C10—H10	106.8	H26A—C26—H26B	109.5
C12—C10—H10	106.8	O9—C26—H26C	109.5
C11—C10—H10	106.8	H26A—C26—H26C	109.5
C17—C11—C1	115.88 (14)	H26B—C26—H26C	109.5
C17—C11—C10	109.17 (14)	O6—C27—O7	124.10 (18)
C1—C11—C10	107.62 (14)	O6—C27—C28	125.78 (18)
C17—C11—C5	98.85 (13)	O7—C27—C28	110.12 (15)
C1—C11—C5	113.26 (14)	C33—C28—C29	119.70 (18)
C10—C11—C5	111.93 (14)	C33—C28—C27	119.17 (18)
C13—C12—C10	106.80 (15)	C29—C28—C27	121.11 (17)
C13—C12—H12A	110.4	C30—C29—C28	119.68 (19)
C10—C12—H12A	110.4	C30—C29—H29	120.2
C13—C12—H12B	110.4	C28—C29—H29	120.2
C10—C12—H12B	110.4	C31—C30—C29	120.2 (2)
H12A—C12—H12B	108.6	C31—C30—H30	119.9
O10—C13—C14	112.75 (15)	C29—C30—H30	119.9
O10—C13—C12	110.67 (15)	C32—C31—C30	120.1 (2)
C14—C13—C12	102.56 (15)	C32—C31—H31	120.0
O10—C13—C16	110.00 (15)	C30—C31—H31	120.0
C14—C13—C16	110.15 (15)	C31—C32—C33	120.0 (2)
C12—C13—C16	110.52 (15)	C31—C32—H32	120.0
O7—C14—C13	108.62 (15)	C33—C32—H32	120.0
O7—C14—C9	114.88 (15)	C28—C33—C32	120.3 (2)
C13—C14—C9	101.87 (14)	C28—C33—H33	119.9
O7—C14—H14	110.4	C32—C33—H33	119.9
			
C21—O3—C1—C2	80.91 (18)	C27—O7—C14—C9	−71.7 (2)
C21—O3—C1—C11	−151.14 (15)	O10—C13—C14—O7	−74.30 (18)
O3—C1—C2—C3	165.95 (14)	C12—C13—C14—O7	166.65 (14)
C11—C1—C2—C3	42.5 (2)	C16—C13—C14—O7	48.99 (18)
C1—C2—C3—C4	−53.6 (2)	O10—C13—C14—C9	164.07 (14)
C2—C3—C4—C18	−175.02 (15)	C12—C13—C14—C9	45.02 (16)
C2—C3—C4—C19	−55.48 (19)	C16—C13—C14—C9	−72.64 (17)
C2—C3—C4—C5	64.08 (19)	C8—C9—C14—O7	−46.2 (2)
C18—C4—C5—C6	69.60 (19)	C10—C9—C14—O7	−165.74 (14)
C3—C4—C5—C6	−173.13 (15)	C8—C9—C14—C13	71.04 (17)
C19—C4—C5—C6	−51.42 (18)	C10—C9—C14—C13	−48.54 (16)
C18—C4—C5—C11	−177.36 (14)	O4—C8—C15—O8	105.06 (16)
C3—C4—C5—C11	−60.09 (18)	C7—C8—C15—O8	−16.5 (2)
C19—C4—C5—C11	61.63 (18)	C9—C8—C15—O8	−143.62 (15)
C23—O2—C6—C7	117.1 (8)	O4—C8—C15—C16	−129.25 (15)
C23'—O2—C6—C7	139.0 (2)	C7—C8—C15—C16	109.20 (17)
C23—O2—C6—C5	−123.4 (8)	C9—C8—C15—C16	−17.9 (2)
C23'—O2—C6—C5	−101.5 (3)	C26—O9—C16—C13	−166.15 (16)
C4—C5—C6—O2	−26.0 (2)	C26—O9—C16—C15	69.0 (2)
C11—C5—C6—O2	−142.99 (15)	O10—C13—C16—O9	33.85 (19)
C4—C5—C6—C7	96.14 (16)	C14—C13—C16—O9	−91.03 (17)
C11—C5—C6—C7	−20.81 (16)	C12—C13—C16—O9	156.36 (15)
O2—C6—C7—C8	−125.94 (15)	O10—C13—C16—C15	155.52 (15)
C5—C6—C7—C8	107.33 (15)	C14—C13—C16—C15	30.6 (2)
O2—C6—C7—C17	115.01 (15)	C12—C13—C16—C15	−81.97 (19)
C5—C6—C7—C17	−11.72 (17)	O8—C15—C16—O9	−96.10 (17)
C24—O4—C8—C7	69.82 (19)	C8—C15—C16—O9	135.47 (16)
C24—O4—C8—C9	−176.53 (15)	O8—C15—C16—C13	144.07 (15)
C24—O4—C8—C15	−57.24 (19)	C8—C15—C16—C13	15.6 (2)
C6—C7—C8—O4	61.11 (17)	C20—N1—C17—C11	172.24 (15)
C17—C7—C8—O4	175.03 (13)	C19—N1—C17—C11	−58.04 (19)
C6—C7—C8—C9	−48.02 (17)	C20—N1—C17—C7	−75.23 (19)
C17—C7—C8—C9	65.90 (18)	C19—N1—C17—C7	54.5 (2)
C6—C7—C8—C15	−177.69 (14)	C1—C11—C17—N1	−52.12 (19)
C17—C7—C8—C15	−63.77 (19)	C10—C11—C17—N1	−173.77 (14)
O4—C8—C9—C14	88.71 (16)	C5—C11—C17—N1	69.21 (15)
C7—C8—C9—C14	−157.77 (15)	C1—C11—C17—C7	−174.41 (14)
C15—C8—C9—C14	−26.1 (2)	C10—C11—C17—C7	63.93 (16)
O4—C8—C9—C10	−157.70 (14)	C5—C11—C17—C7	−53.09 (15)
C7—C8—C9—C10	−44.17 (18)	C8—C7—C17—N1	167.06 (14)
C15—C8—C9—C10	87.46 (18)	C6—C7—C17—N1	−76.90 (17)
C14—C9—C10—C12	32.56 (16)	C8—C7—C17—C11	−75.30 (16)
C8—C9—C10—C12	−87.55 (16)	C6—C7—C17—C11	40.75 (16)
C14—C9—C10—C11	160.74 (15)	C22—O1—C18—C4	−178.75 (16)
C8—C9—C10—C11	40.6 (2)	C3—C4—C18—O1	−69.66 (18)
O3—C1—C11—C17	−51.58 (19)	C19—C4—C18—O1	169.40 (14)
C2—C1—C11—C17	71.3 (2)	C5—C4—C18—O1	49.16 (19)
O3—C1—C11—C10	70.91 (17)	C20—N1—C19—C4	173.11 (15)
C2—C1—C11—C10	−166.17 (15)	C17—N1—C19—C4	42.3 (2)
O3—C1—C11—C5	−164.83 (14)	C18—C4—C19—N1	−165.02 (15)
C2—C1—C11—C5	−41.9 (2)	C3—C4—C19—N1	77.44 (18)
C9—C10—C11—C17	−52.69 (19)	C5—C4—C19—N1	−42.49 (19)
C12—C10—C11—C17	68.88 (19)	C8—O4—C24—O5	1.2 (3)
C9—C10—C11—C1	−179.23 (14)	C8—O4—C24—C25	−179.41 (16)
C12—C10—C11—C1	−57.65 (19)	C14—O7—C27—O6	−5.0 (3)
C9—C10—C11—C5	55.70 (19)	C14—O7—C27—C28	175.49 (14)
C12—C10—C11—C5	177.28 (14)	O6—C27—C28—C33	−2.5 (3)
C4—C5—C11—C17	−73.55 (16)	O7—C27—C28—C33	177.03 (18)
C6—C5—C11—C17	45.83 (15)	O6—C27—C28—C29	179.06 (19)
C4—C5—C11—C1	49.66 (18)	O7—C27—C28—C29	−1.4 (2)
C6—C5—C11—C1	169.05 (14)	C33—C28—C29—C30	0.7 (3)
C4—C5—C11—C10	171.55 (13)	C27—C28—C29—C30	179.11 (18)
C6—C5—C11—C10	−69.06 (16)	C28—C29—C30—C31	0.0 (3)
C9—C10—C12—C13	−5.33 (18)	C29—C30—C31—C32	−0.6 (3)
C11—C10—C12—C13	−134.06 (15)	C30—C31—C32—C33	0.6 (3)
C10—C12—C13—O10	−144.52 (15)	C29—C28—C33—C32	−0.7 (3)
C10—C12—C13—C14	−24.02 (18)	C27—C28—C33—C32	−179.21 (19)
C10—C12—C13—C16	93.37 (17)	C31—C32—C33—C28	0.1 (3)
C27—O7—C14—C13	175.00 (15)		

### Hydrogen-bond geometry (Å, °)

**Table:**

*D*—H···*A*	*D*—H	H···*A*	*D*···*A*	*D*—H···*A*
O8—H8···O5	0.84	2.11	2.7887 (19)	137
O10—H10O···O9	0.84	2.05	2.5620 (19)	118
